# Adaptive filters at the first olfactory synapse

**DOI:** 10.1371/journal.pbio.3003405

**Published:** 2025-09-17

**Authors:** Elizabeth H. Moss, Benjamin R. Arenkiel

**Affiliations:** 1 Department of Anesthesiology and Perioperative Medicine, Oregon Health & Science University, Portland, Oregon, United States of America; 2 Department of Neuroscience, Baylor College of Medicine, Houston, Texas, United States of America; 3 Department of Molecular and Human Genetics, Baylor College of Medicine, Houston, Texas, United States of America; 4 Jan and Dan Duncan Neurological Research Institute, Texas Children’s Hospital, Houston, Texas, United States of America

## Abstract

The olfactory system is able to filter odor representations based on attention and learning. This Primer discusses two PLOS Biology studies that reveal how short axon cells in the olfactory bulb integrate cholinergic input from the basal forebrain to dynamically regulate olfactory input.

## Teaser

The olfactory system is able to filter odor representations based on attention and learning. Two *PLoS Biology* studies reveal how short axon cells in the olfactory bulb integrate cholinergic input from the basal forebrain to dynamically regulate olfactory input.

**Fig 1:**
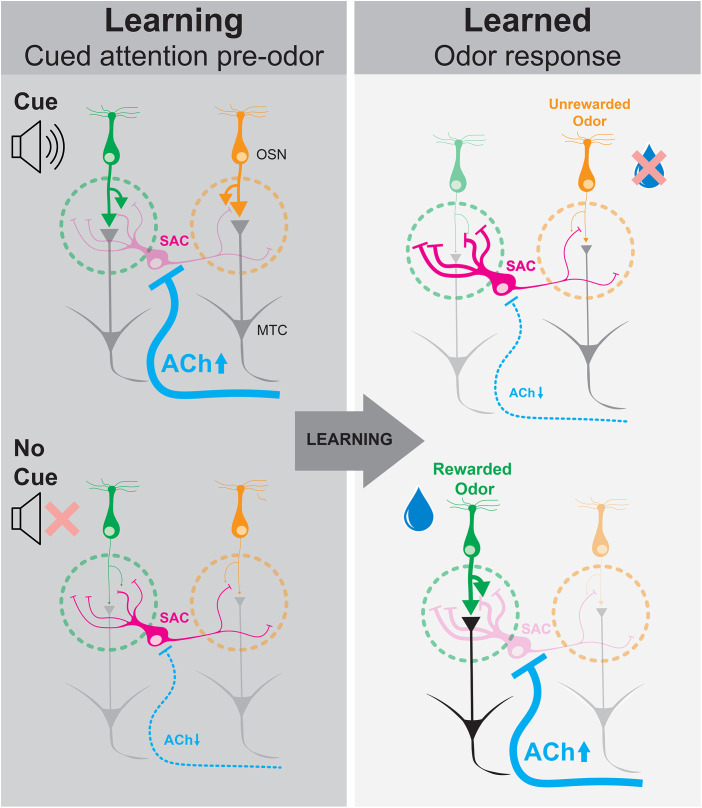
Short axon cells of the olfactory bulb dynamically filter olfactory sensory input during attention and after learning. As mice learn to discriminate odors (left), providing a cue before presenting odors (top) improves performance by recruiting cholinergic signaling from the basal forebrain (ACh, blue) to inhibit short axon cells (SAC, magenta) which, in turn, disinhibits olfactory sensory neuron (OSN, green and orange) axon terminals in olfactory bulb glomeruli (dashed circles), and increases their signaling onto mitral and tufted cells (MTCs, gray). After learning (right), SACs remodel to make stronger contacts with reward-associated OSNs (top) and cholinergic signaling is disengaged during the cued period before odor presentations. Cholinergic signaling, however, is strongly recruited during presentations of the reward-associated odor (bottom), allowing disinhibition of reward-linked odor signaling from OSNs to MTCs.

All sensory systems confront the same challenge: spotlighting information that matters, while ignoring a flood of distractions. In vision, audition, and smell alike, attention and learning are critical for this filtering, but the underlying circuit mechanisms remain largely unknown. In this issue of *PLoS Biology*, two companion studies by Garg and colleagues [1,2] reveal a mechanism in the mouse olfactory bulb (OB) that implements attention and learning-related filtering of sensory input. Together, these studies uncover how local interneurons called short axon cells (SACs) serve as a hub where bottom-up sensory drive and top-down attentional modulation converge to refine odor information as it first enters the brain (Fig 1).

The OB is the brain’s gateway for smell. In the OB, axons from olfactory sensory neurons (OSNs) converge onto discrete glomerular structures based on the odorant receptors they express. Thus, each glomerulus represents a distinct channel of information, providing a spatial “map” of odor identity [3,4]. But glomeruli are not simple relays. They are embedded in a rich network of inhibitory interneurons that sculpt how signals are transmitted to downstream circuits. While most interneurons in the glomerular layer of the OB provide local, glomerulus-specific inhibition, SACs spread their processes across multiple glomeruli where they synapse directly onto OSN terminals as well as onto other interneurons [5–8]. Thus, they are uniquely positioned to compare, contrast, and redistribute activity across the glomerular map. Much like lateral inhibition in the retina sharpens visual signals by enhancing contrast between neighboring photoreceptors, SACs sharpen olfactory signals by shaping activity patterns across neighboring glomeruli. This arrangement gives SACs outsized influence over the very first step in odor processing, effectively tuning and filtering sensory signals as they enter the OB.

Odor response maps are not only shaped by incoming sensory signals but also by descending projections from higher brain regions. Prominent among these are cholinergic inputs from the basal forebrain horizontal limb of the diagonal band (HDB). In practical terms, top-down cholinergic input works like a switch that can activate or deactivate filters, sometimes letting all the light through, other times engaging filters to sharpen contrast or block out glare depending on what you want to focus on. Here, Garg and colleagues leverage the role of cholinergic modulation in arousal, learning, and attention to resolve how it influences sensory processing by dynamically engaging filters within the OB.

The first study by Garg and colleagues [1] demonstrates how the top-down cholinergic pathway operates during attention. To trigger heightened attention, they trained mice to discriminate odors after an auditory cue—asking mice to report which of two smells was presented by licking a spout for a water reward. As mice learned to discriminate odors, HDB cholinergic neurons were strongly activated in the time period between the cue and odor delivery. This cholinergic activation suppressed SAC firing via muscarinic receptor signaling, reducing inhibition onto OSN terminals and allowing stronger, sharper responses to rewarded odors and boosting decision accuracy.

Causal manipulations confirmed the circuit logic. Inhibition of SACs between the cue and odor delivery mimicked the attentional effect of the cue, while manipulations during odor presentations had little effect. Likewise, silencing HDB cholinergic neurons abolished the behavioral improvement provided by the cue. These results show how SACs act as a critical intermediary in odor processing, translating global attentional signals into specific, glomerulus-level modulation of odor responses.

The companion paper [2] zooms in on SACs themselves, showing how they transform a global attentional cue into a sharp, specific filter for reward-related odor input. The authors show that SACs undergo selective plasticity after discriminative training, with tyrosine hydroxylase, the rate-limiting enzyme for dopamine synthesis, upregulated in SACs surrounding glomeruli activated by rewarded odors. Furthermore, genes associated with synaptic plasticity were enriched, and electron microscopy revealed an increase in physical contacts between SACs and OSN terminals. These cellular changes were paralleled by functional changes, where glomeruli became more strongly selective for the rewarded odor after training.

Together, the two studies outline a mechanism for adaptive filtering and stimulus learning at the first synapse of the olfactory system. With experience, SAC connectivity is remodeled so that rewarded OSN inputs are preferentially enhanced even without an ongoing top-down drive. Computational modeling captures this two-phase operation: combining cholinergic modulation with activity-dependent plasticity at SAC–OSN connections reproduces both the physiological data and the behavioral shift from cue-dependent to automatic performance.

These findings elevate SACs to central computational players in OB circuits. By integrating bottom-up and top-down signals, they allow the system to flexibly deploy attention when needed and to hardwire selectivity through learning. Yet key questions remain. For instance, do SACs use co-transmission for these functions? SACs can release both dopamine and GABA, but the present studies do not resolve whether dopamine, GABA, or their interplay shape behavior and circuit dynamics, nor whether dopaminergic signaling itself is spatially or temporally tuned by attention and learning. Also, OSNs and SACs are reciprocally connected. After learning-related structural remodeling, is connectivity enhanced bidirectionally or is one direction favored leading to imbalanced OSN excitation of SACs and SAC reciprocal inhibition of OSNs? Disentangling reciprocal connectivity and distinct contributions of dopamine versus GABA through direct manipulations of transmitter release and/or receptor signaling will be essential to reveal how this circuit transforms raw sensory input into a selectively filtered signal at the very first synapse.

These two studies clearly establish SACs as a central hub where attention and learning converge at the synaptic level, but they also open the door to exciting future directions. Given that the current work highlights how cholinergic recruitment of SACs can sharpen odor representations and that SAC plasticity can bias input toward rewarded stimuli, a future challenge will be to determine how this core motif operates in more complex situations, for example, when odors are similar, ambiguous, or when multiple cues must be tracked at once. Moreover, can this filter be acutely tuned on a moment-to-moment basis? Answering these questions will reveal whether SACs operate mainly as a slowly reconfigurable circuit element or as a highly dynamic filter that can flexibly adjust to shifting sensory demands. In this way, the disinhibitory HDB–SAC motif defined here sets the stage for future work to uncover the full range of strategies that sensory systems use to meet challenges of real-world perception. By showing how attention and learning converge on a single interneuron type at the gateway to the olfactory system, these studies not only pinpoint cellular players and their circuit impact but also open the door to a broader principle of neural circuit function: an adaptive filtering strategy that sensory systems may use across the brain.

## Abbreviations

HDB: horizontal limb of the diagonal band
MTCs: mitral and tufted cells
OB: olfactory bulb
OSN: olfactory sensory neuron
SACs: short axon cells
